# Artificial intelligence for predicting pathological complete response to neoadjuvant therapy in triple-negative breast cancer: A systematic review and meta-analysis

**DOI:** 10.1016/j.breast.2026.104861

**Published:** 2026-07-02

**Authors:** Andrea Carlini, Flavia Foca, Marianna Sirico, Alberto Farolfi, Chiara Casadei, Caterina Gianni, Michela Palleschi, Nicola Gentili, Marita Mariotti, Giandomenico Di Menna, Alice Andalò, Paolo De Angelis, Olga Serra, Simone Sabbioni, Giulia Miserocchi, Daniela Montanari, Lorenzo Cecconetto, Samanta Sarti, Antonino Musolino, Filippo Merloni

**Affiliations:** a Department of Medical and Surgical Sciences, University of Bologna, Bologna, Italy; b Biostatistics and Clinical Trial Unit, IRCCS Istituto Romagnolo per lo Studio dei Tumori (IRST) "Dino Amadori', Meldola, FC, Italy; c Breast & GYN Unit, Medical Oncology, IRCCS Istituto Romagnolo per lo Studio dei Tumori (IRST) “Dino Amadori”, Meldola, (FC), Italy; d Data Unit & Outcome Research, IRCCS Istituto Romagnolo per lo Studio dei Tumori (IRST) "Dino Amadori', Meldola, (FC), Italy; e Department of Medicine and Surgery, University of Parma, Parma, Italy

**Keywords:** Triple-negative breast cancer, Neoadjuvant therapy, Artificial intelligence, Pathological complete response, Meta-analysis

## Abstract

**Background:**

Artificial intelligence (AI) has emerged as a promising approach to predict pathological complete response (pCR) to neoadjuvant systemic therapy (NAST) in early triple-negative breast cancer (eTNBC), but the available evidence remains methodologically heterogeneous and has not been quantitatively synthesized.

**Methods:**

We conducted a systematic review and meta-analysis of studies evaluating AI-based models for pCR prediction after NAST in eTNBC. PubMed/MEDLINE, Embase, and CENTRAL were searched up to January 1, 2026. Eligible studies included original investigations applying machine learning or deep learning to radiological, pathological, clinical, or omics data, with pCR discrimination reported at least as area under the receiver operating characteristic curve (AUC). Random-effects meta-analysis of logit-transformed AUCs was performed on studies with sufficient data to reconstruct AUC variance.

**Results:**

Fifty-eight studies were included in the systematic review, and 20 cohorts from 19 studies were eligible for quantitative synthesis. Overall, AI-based models showed high discriminative performance for predicting pCR, with a pooled AUC of 0.79 (95% CI, 0.75–0.83). However, substantial heterogeneity was observed (I^2^ = 73.1%). Models incorporating radiomics and longitudinal data showed numerically higher AUCs than non-radiomics and baseline-only approaches, respectively, although subgroup differences were not statistically significant. Multimodal models did not significantly outperform unimodal models. Funnel plot asymmetry suggested possible small-study effects or publication bias**.**

**Conclusions:**

AI-based models demonstrate promising accuracy for predicting pCR after NAST in eTNBC, but their clinical applicability is limited by substantial heterogeneity, inconsistent validation strategies, and potential publication bias. Prospective multicenter studies with standardized reporting and robust external validation are mandatory before routine clinical implementation.

## Highlights

• AI models predict pCR after NAST in early triple-negative breast cancer.
• Meta-Analysis of 20 cohorts showed a pooled AUC of 0.79.
• Radiomics and longitudinal data showed the highest predictive accuracy.
• Multimodal AI models did not significantly outperform unimodal approaches.
• Clinical implementation requires external validation and standardization.

## Graphical abstract

**Figure:**
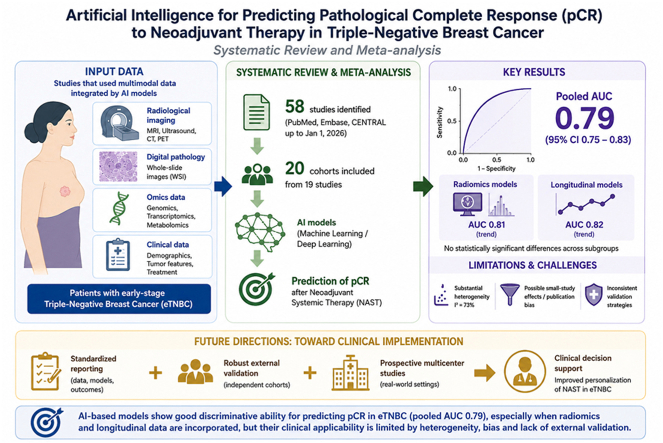

## 1 Introduction

Triple-negative breast cancer (TNBC) accounts for approximately 15-20% of all breast cancers and is historically defined by the absence of estrogen receptor (ER), progesterone receptor (PR) and HER2 expression [1,2]. This disease subtype is typically associated with aggressive biological behavior, high proliferative index, and poor prognosis [3,4]. Because of the lack of endocrine or HER2-targeted treatment options, systemic chemotherapy and immunotherapy remain the cornerstone of treatment for early-stage TNBC (eTNBC), defined as stage I–III disease according to the American Joint Committee on Cancer (AJCC) TNM classification. In this context, neoadjuvant systemic therapy (NAST) plays a pivotal role, offering the opportunity not only to downstage tumors and facilitate breast-conserving surgery, but also to provide early information about treatment sensitivity [3,[5], [6], [7]]. The achievement of a pathological complete response (pCR)—defined as the absence of residual invasive disease in the breast and axillary lymph nodes (ypT0/is, ypN0)—has consistently been shown to correlate with improved disease-free and overall survival, particularly in TNBC and HER2-positive subtypes [8,9].

Despite its prognostic value, the rate of pCR remains variable across patients, and reliable tools to predict individual response are still lacking. Conventional predictive models, largely based on clinical and biological parameters (e.g. patient age, tumor size, nodal status, histological grade, proliferation markers etc.) have demonstrated limited accuracy [10,11]. Artificial intelligence (AI), including both machine learning (ML) and deep learning (DL) algorithms, has emerged as a transformative technology in the breast cancer (BC) field [12,13]. ML is a branch of AI that enables algorithms to learn patterns from data and make predictions or decisions. DL, a specialized subset of ML, uses multi-layered neural networks to automatically extract complex features from large datasets. In medical imaging, radiomics applies ML and DL techniques to quantitative features extracted from radiological images, uncovering patterns that may not be visible to the human eye. Similarly, computational pathology leverages ML and DL methods to analyze digitized histopathological images, enabling automated feature extraction, disease characterization, and predictive modeling. Together, radiomics and computational pathology have been proposed to capture subtle phenotypic characteristics from imaging or pathology data that are invisible to the human eye, supporting precision medicine and improve clinical decision-making [14,15]. Simultaneously, advancements in genomics, transcriptomics, and metabolomics are broadening the predictive landscape, facilitating the integration of molecular tumor signatures with clinical and imaging variables [16]. This strategy, known as the multimodal approach, is increasingly recognized as a powerful way to comprehensively capture the complexity of tumor biology and predict treatment response.

Several systematic reviews have highlighted the expanding role of AI in BC care, encompassing screening, diagnosis, treatment planning, and response monitoring [17]. Within this landscape, *Krasniqi* et al. reviewed the emerging evidence on multimodal DL models for predicting pCR following NAST, reporting median area under the curve (AUC) values approaching 0.88 [18]. Nonetheless, the literature is characterized by marked methodological heterogeneity, a predominance of retrospective designs, small sample sizes, and limited external validation. Collectively, these factors constrain generalizability and underscore the need for a rigorous quantitative synthesis and standardized comparison across AI methodologies.

Given this context, the objective of this systematic review and meta-analysis is to comprehensively evaluate the role of AI in predicting response to NAST in eTNBC. Specifically, we aim to (i) synthesize the available evidence and provide a quantitative estimate of AI predictive performance using AUC as the primary endpoint; (ii) compare the performance of different AI methodologies and data modalities; (iii) assess the relative contribution of unimodal versus multimodal frameworks; and (iv) investigate potential sources of variability in model performance, including data type, timing of acquisition, and methodological characteristics. By integrating quantitative synthesis with methodological analysis, this work aims to clarify the current performance and limitations of AI models in this setting and to inform future research toward more robust, generalizable, and clinically applicable predictive tools.

## 2 Materials and methods

This systematic review and meta-analysis was designed and reported in accordance with the Preferred Reporting Items for Systematic Reviews and Meta-Analyses (PRISMA) recommendations. The methods used for searching, selecting studies, extracting data, and performing the quantitative synthesis are outlined below.

### 2.1 Literature search strategy

A comprehensive literature search was performed in PubMed/MEDLINE, Embase, and Cochrane Central Register of Controlled Trials (CENTRAL) databases, covering all publications up to 1st January 2026. The search aimed to identify studies applying AI methodologies to predict NAST response in patients with eTNBC. The strategy was developed using a structured combination of controlled vocabulary terms and free-text keywords grouped into three conceptual domains: (1) breast cancer ("breast cancer', "breast carcinoma', "breast neoplasm', and related synonyms); (2) neoadjuvant treatment ("neoadjuvant therapy', "neoadjuvant chemotherapy', "neoadjuvant immunotherapy', "preoperative treatment', "primary systemic therapy', and related synonyms); and (3) artificial intelligence ("artificial intelligence', "machine learning', "deep learning', "radiomics', "computational pathology', "omics', "transformers', "large language models', and related subfields). These domains were combined using the Boolean operator AND, and the search syntax was adapted to the specific indexing systems of each database. Only articles published in English were considered, while no geographical restrictions were applied. In addition, reference lists of all included articles and relevant systematic reviews were manually screened to identify additional eligible studies. The full search strategy and detailed query syntax for each database are provided in the Supplementary Materials (Supplementary Table 1).

### 2.2 Study eligibility criteria

Studies were considered eligible if they met all of the following conditions: (1) original prospective or retrospective investigations; (2) inclusion of patients with eTNBC treated with NAST, regardless of the therapeutic regimen administered; (3) evaluation of pCR, defined as the absence of residual invasive disease in both the breast and axillary lymph nodes (ypT0/is ypN0), as the pathological response endpoint; (4) application of AI-based analytical methods (ML or DL) to at least one data modality relevant for predicting pCR, including radiological imaging, histopathology, genomic or metabolomic data, and/or clinical variables; (5) reporting of at least one measure of predictive discrimination, with the AUC considered the minimum requirement; and (6) inclusion of mixed BC subtypes only when TNBC-specific outcomes could be extracted separately. Studies were excluded in cases where TNBC-specific results were not separable from other molecular subtypes, the pathological response endpoint was limited to axillary nodal response only, non-standard pathological endpoints were reported without extractable pCR data, or AI-based analytical methods were absent. Additionally, case reports, conference abstracts, editorials, meta-analyses, systematic reviews, and narrative reviews were excluded.

### 2.3 Study selection and data collection

Two authors (AC and FM) independently screened all titles and abstracts retrieved through the literature search, followed by full-text evaluation of potentially eligible studies. Disagreements were resolved by discussion, and when consensus could not be reached, a third reviewer was consulted. For each included study, data were extracted into a standardized database capturing the following variables: first author and year of publication, study design, single-center or multicenter setting, size of the training and validation cohorts, patient and tumor characteristics, NAST regimen, AI methodology (classical ML versus DL; unimodal versus multimodal), type of input data (radiological, pathological, genomic/metabolomic, or clinical), validation strategy (internal cross-validation, split-sample validation, or external validation), frequency of pCR, and predictive performance metrics. The AUC was considered the primary outcome, while secondary metrics such as sensitivity, specificity, and accuracy were collected when available. Data collection was performed independently by two reviewers (AC and FF), with discrepancies resolved by consensus.

### 2.4 Statistical methods for descriptive and quantitative analysis

Categorical variables were expressed as percentages, whereas continuous variables were summarized as median and interquartile range (IQR). Differences between studies included in the meta-analysis and those excluded were assessed using the chi-square test or Fisher exact test as appropriate, in order to explore potential selection bias. The primary outcome was the discriminatory performance of AI models for predicting pCR, expressed as the AUC from the internal validation cohort, which represents the most consistently reported and comparable metric across studies. When multiple performance estimates were reported within the same study, the AUC corresponding to the final internally validated model, as identified by the study authors, was extracted. Training-set performance was not considered. For studies reporting at least one internal validation cohort, variability measures were reconstructed from the reported 95% confidence intervals (CIs) of the AUC. For studies not reporting directly the 95% CIs, the variance of the AUC was estimated using the method described by Hanley and McNeil [19]. Studies for which insufficient information was available to estimate AUC variability, as well as studies including fewer than 30 patients in the validation cohort, were excluded from the quantitative analysis. AUC values were transformed using the logit function and random-effects meta-analysis was conducted using restricted maximum likelihood estimation from the *metafor* package in R. Pooled estimates were back-transformed to the AUC scale using the inverse-logit transformation to enhance interpretability. Between-study heterogeneity was quantified using the between-study variance (τ^2^, on the logit scale) and the inconsistency statistic (I^2^). Forest plots were generated on the AUC scale via inverse-logit back-transformation. Radial (Galbraith) and Baujat plots were used to explore potential outliers and influential studies. Small-study effects were assessed through visual inspection of funnel plots and formally tested using an Egger-type regression test. Pre-specified subgroup analyses were conducted to explore potential sources of heterogeneity, including comparisons between radiomics versus non-radiomics approaches, computational pathology versus non-computational pathology models, genomic/metabolomic versus non-genomic/metabolomic methods, unimodal versus multimodal AI strategies, and differences according to the type of AI approach used. Each categorical variable was included as a moderator in mixed-effects models, and subgroup differences were assessed using omnibus tests of moderators. Meta-regression analyses were performed to evaluate the proportion of between-study heterogeneity explained by individual moderators. The proportion of explained heterogeneity was quantified using a pseudo-R^2^ statistic, defined as the relative reduction in τ^2^ compared with the corresponding null model. The risk of bias and applicability of the included studies were assessed using the Prediction model Risk Of Bias ASsessment Tool (PROBAST) [20]. Although several included studies employed ML and radiomics approaches, many models were based on conventional statistical or hybrid methods and did not involve highly complex AI pipelines. PROBAST evaluates four domains for risk of bias (participants, predictors, outcome, and analysis) and three domains for applicability (participants, predictors, and outcome). Two reviewers independently evaluated each study, and disagreements were resolved by consensus. All analyses were conducted in R (R Foundation for Statistical Computing, version 4.4.2).

## 3 Results

### 3.1 Study selection

**Fig. 1:**
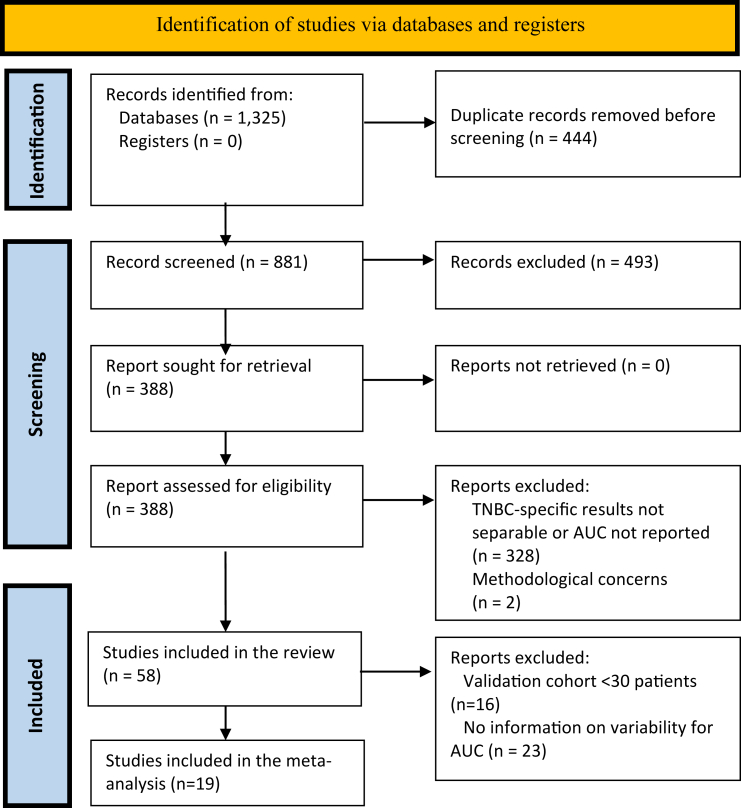
PRISMA flowchart illustrating the study selection process.

A total of 1325 records were identified through the comprehensive search of PubMed/MEDLINE, Embase, and Cochrane CENTRAL databases. After removal of duplicates, 493 records were excluded because they were non-pertinent, did not involve AI-based methodologies, or did not represent original research articles. Of the remaining records, 328 studies were excluded after title/abstract and full-text screening because TNBC-specific results could not be isolated for analysis or because predictive performance metrics required for quantitative analysis—particularly the AUC—were not reported. Two studies by Zhou et al. were subsequently excluded because they represented duplicate analyses of the same dataset and raised methodological concerns regarding independence between testing and internal validation cohorts [21,22]. Therefore, 58 studies were retained in the final set of included studies [23].– [24] Among these, studies providing sufficient information to estimate AUC and its variability were incorporated into the meta-analysis, as described below. The selection process is shown in Fig. 1.

### 3.2 Characteristics of included studies

**Fig. 2:**
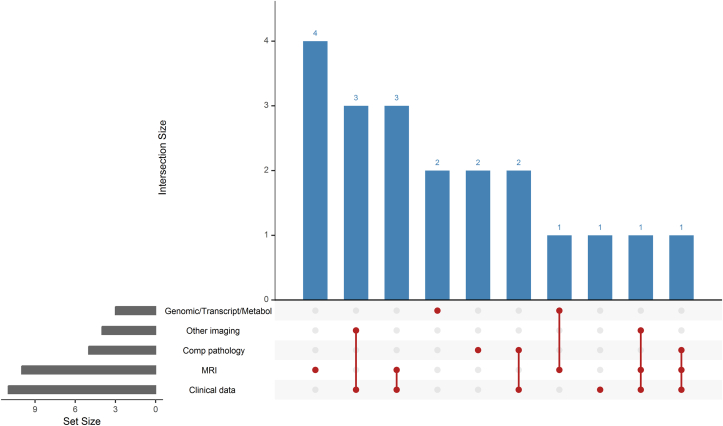
UpSet plot showing the frequency and overlap of data modalities used across studies. Horizontal bars represent the total number of studies using each modality (set size), while vertical bars represent the number of studies using the specific combinations indicated by the connected dots below (intersection size).

The 58 included studies were published between 2018 and 2025 and were predominantly retrospective in design (n = 52; 89.7%) [23]– [25,26]– [27,28]– [29,30]– [[31], [32], [33], [34]]– [24], with only a small minority being prospective (n = 4; 6.9%) [[35], [36], [37], [38]] or mixed investigations (n = 2; 3.4%) [39,40]. Internal validation procedures were reported in 45 studies (77.9%) [23]– [[41], [42], [43], [44]]– [27,28,45]– [30,39]– [31,33,38,46]– [47,48]– [49,50]– [24,51,52], most commonly through cross-validation or split-sample approaches, while a formal split between training and validation sets was explicitly described in 28 studies (73.7%) [25,29,30,[33], [34], [35],^37^,^39^,^40^,^42^,^43^,^46^,[48], [49], [50], [51], [52], [53], [54], [55], [56], [57], [58], [59], [60], [61], [62], [63]]. External validation using an independent cohort was performed in 22 studies (37.9%) [[23], [24], [25],^27^,^31^,^34^,^36^,[38], [39], [40],^43^,^46^,^47^,^55^,^59^,^62^,[64], [65], [66], [67], [68], [69]]. Sample sizes varied substantially across studies, with a median of 74.5 patients (interquartile range [IQR]: 45.25–112) in the training cohorts, 48 patients (IQR: 20–86.75) in internal validation cohorts and 64 patients (IQR: 41–106.5) in external validation cohorts. 33 studies (56.9%) included mixed molecular subtypes [[23], [24], [25],^27^,^29^,^31^,[33], [34], [35],^39^,^40^,^42^,^45^,^50^,^51^,[53], [54], [55],^58^,^59^,^61^,^62^,[65], [66], [67], [68], [69], [70], [71], [72], [73], [74], [75]], but reported separate analyses for the TNBC cohort, whereas 25 studies (43.1%) focused exclusively on patients with eTNBC [26,28,30,32,[36], [37], [38],^41^,^43^,^44^,[46], [47], [48], [49],^52^,^56^,^57^,^60^,^63^,^64^,[76], [77], [78], [79], [80]]. Consistent with the eligibility criteria, all included studies evaluated pCR as the pathological response endpoint, defined as the absence of residual invasive disease in both the breast and axillary lymph nodes (ypT0/is ypN0). DL methodologies were used in 12 studies (20.7%) [44,53 31,32,[34], [35], [36], [37],^39^,^50^,^63^,^80^], whereas classical ML approaches remained prevalent, appearing in 46 studies (79.3%) [23]– [43,72]– [25,26]– [36,65]– [29,73]– [33,38,40,47,74]– [51,67,69,75]– [24]. The most commonly used ML algorithms included logistic regression (LR; n = 26) [25,26,29,31,33,[36], [37], [38], [39], [40],^43^,^45^,^49^,^52^,^54^,^57^,^59^,^61^,^62^,^64^,^65^,[69], [70], [71],^73^,^78^], support vector machines (SVM; n = 19) [26,27,29,30,37,[40], [41], [42],^51^,^54^,^59^,^62^,^66^,^67^,[73], [74], [75], [76],^78^], random forests (RF; n = 14) [26,29,30,40,46,51,54,[59], [60], [61], [62],^72^,^73^,^78^], least absolute shrinkage and selection operator (LASSO; n = 7) [31,48,61,67,73,76,78], and extreme gradient boosting (XGBoost; n = 7) [27,28,43,51,54,59,61]. With respect to input data modalities, imaging-derived data were utilized in 37 studies (63.8%) [[24], [25], [26],[29], [30], [31],[33], [34], [35], [36], [37], [38], [39],^42^,^45^,[47], [48], [49],[51], [52], [53], [54], [55],^58^,^59^,^62^,^64^,^65^,^67^,[70], [71], [72],[74], [75], [76],^78^,^79^], computational pathology approaches in 15 studies (31.0%) [23,32,[39], [40], [41],^43^,^44^,^50^,^56^,^63^,^66^,^68^,^69^,^77^,^80^], and clinical variables in 37 studies (63.8%) [23,[25], [26], [27],^29^,^31^,[33], [34], [35], [36],[38], [39], [40], [41], [42], [43],^45^,^49^,[53], [54], [55], [56], [57],^59^,^61^,[65], [66], [67], [68], [69],^71^,^73^,^75^,^78^,^80^]. Given the methodological heterogeneity of pathology-based studies, computational pathology approaches are further detailed in the Supplementary Table 2. Genomic, transcriptomic, or metabolomic data were incorporated in 11 studies (19.0%) [28,46,48,49,53,57,60,64,72,78], while longitudinal data acquired during the course of NAST were used in 17 studies (29.3%) [25,27,33,34,36,37,48,52,54,55,59,60,62,64,65,72,76]. Magnetic resonance imaging (MRI) represented the most frequently used data source (n = 26) [26,27,29,30,[36], [37], [38], [39],[47], [48], [49],^51^,^53^,^55^,^58^,^59^,^62^,^64^,^67^,[70], [71], [72],[74], [75], [76],^79^], followed by digital pathology (n = 18) [23,32,[39], [40], [41],^43^,^44^,^50^,^56^,^63^,^64^,^66^,^68^,^69^,[77], [78], [79], [80]], ultrasound (US; n = 7) [25,34,35,42,52,54,65], and other imaging techniques including positron emission tomography (PET) [78], computed tomography (CT) [26,31,33], or mammography [55] (n = 5). Multimodal or hybrid AI frameworks were employed in 34 studies (58.6%) [[25], [26], [27],^29^,^31^,[33], [34], [35],[38], [39], [40],^42^,^43^,^45^,^48^,^49^,[52], [53], [54], [55], [56], [57],[64], [65], [66], [67], [68], [69],^71^,^75^,[78], [79], [80]], integrating complementary data sources to enhance predictive performance. Among these, the majority (n = 26) combined two data modalities [[24], [25], [26], [27],^29^,^31^,[33], [34], [35],^38^,^40^,^43^,^45^,^48^,^52^,^54^,^56^,^57^,^65^,^66^,^68^,^69^,^71^,^75^,^79^,^80^], while eight studies incorporated three or more data sources for model training and validation [24,39,42,49,53,55,64,78]. A descriptive analysis was carried out on studies included in quantitative analysis and Fig. 2 shows that, in this subgroup, clinical and MRI data are the most commonly used modalities, both individually and in combination. A comprehensive overview of study characteristics is provided in Table 1.

**Table 1:** Summary of included studies according to AI methodology, data source type, and main study characteristics.

Variable	Studies included in quantitative analysis		Overall, N = 58 (%)	p-value
	No, N = 39 (%)	Yes, n = 19 (%)		
*Year of publication*				
2018	0 (0.0)	1 (5.3)	1 (1.7)	0.151
2019	1 (2.6)	1 (5.3)	2 (3.4)	
2020	0 (0.0)	0 (0.0)	0 (0.0)	
2021	7 (18.0)	0 (0.0)	7 (12.1)	
2022	9 (23.0)	3 (15.8)	12 (20.7)	
2023	7 (18.0)	3 (15.8)	10 (17.2)	
2024	9 (23.0)	9 (47.4)	18 (31.1)	
2025	6 (15.4)	2 (10.5)	8 (13.8)	
*Study design*				
Mixed	2 (5.1)	0 (0.0)	2 (3.4)	0.634
Retrospective	34 (89.8)	17 (89.5)	52 (89.7)	
Prospective	2 (5.1)	2 (10.5)	4 (6.9)	
*Validation cohort composition*				
All breast cancer subtypes	26 (66.7)	7 (36.8)	33 (56.9)	0.031
TNBC only	13 (33.3)	12 (63.2)	25 (43.1)	
*Information on NAST regimen*	31 (79.5)	12 (63.2)	43 (74.1)	0.213
*Presence of cross-validation*	26 (76.5)	14 (82.4)	40 (80.0)	0.468
Unknown	5	3	8	
*Presence of split of cases*	18 (72.0)	10 (76.9)	28 (73.7)	1.000
Unknown	14	6	20	
*Presence of external validation*	15 (39.5)	7 (36.8)	22 (38.6)	0.847
Unknown	1	0	1	
*Utilization of radiomics*	25 (64.1)	12 (63.2)	37 (63.8)	0.944
*Utilization of computational pathology*	11 (28.2)	4 (21.1)	15 (25.9)	0.752
*Utilization of clinical data*	27 (69.2)	10 (52.6)	37 (63.8)	0.254
*Utilization of genomic data*	8 (20.5)	3 (15.8)	11 (19.0)	1.000
*Combination of radiomics and clinical data*	17 (43.6)	7 (36.8)	24 (41.4)	0.624
*Utilization of longitudinal data*	11 (28.2)	6 (31.6)	17 (29.3)	0.791
*AI design*				
Multimodal	25 (64.1)	9 (47.4)	34 (58.6)	0.225
Unimodal	14 (35.9)	10 (52.6)	24 (41.4)	
*AI modality*				
Classical ML	29 (74.4)	17 (89.5)	46 (79.3)	0.302
DL ( ± ML)	10 (25.6)	2 (10.5)	12 (20.7)	

Of the 58 studies included in the review, 23 were excluded from quantitative analysis because AUC variability could not be reconstructed (absence of standard error, confidence intervals, or sufficient data).[26]–[29,32,37]–[39,41,42,45,47,48,50]–[52,59,67,71,74,77,78,80] Finally, 16 cohorts were excluded because the validation sample size was smaller than 30 patients [30,33,34,[39], [40], [41],^45^,[49], [50], [51],[53], [54], [55],^65^,^72^,^75^]. After these sequential exclusions, 20 cohorts remained eligible for quantitative synthesis deriving from 19 studies [23,26,27,29,31,32,37,38,42,43,[46], [47], [48],^52^,^58^,^60^,^61^,^76^,^77^]. As shown in Table 1, comparison of study characteristics between included and excluded studies identified differences in population composition, with included studies more frequently focusing on TNBC and excluded studies more often including mixed histological subtypes (p-value: 0.031).

### 3.3 Quantitative analysis of predictive performance

**Fig. 3:**
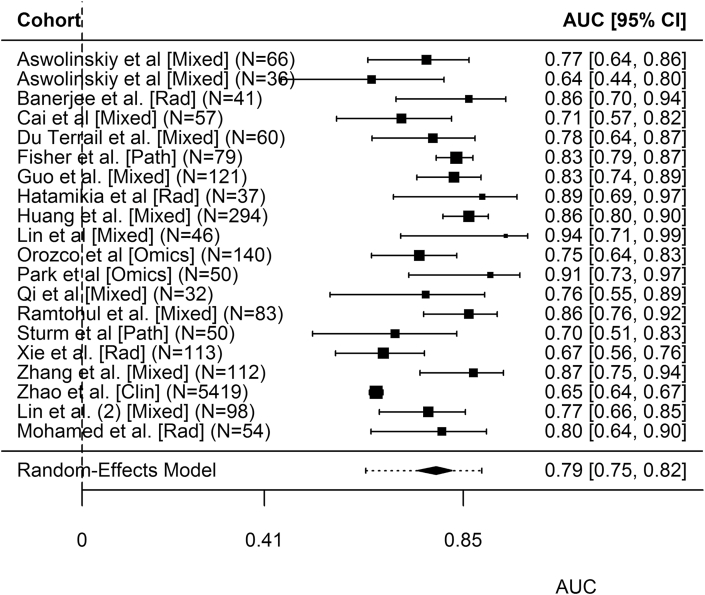
Forest plot for studies included in meta-analysis. [Rad]: Radiological; [Path]: Computational pathology; [Omics]:Genomics/Transcriptomics/Metabolomics; [Clin]:Clinical data; [Mixed]:Two or more modalities.

Overall, the pooled AUC of AI-based models for predicting pCR following NAST was 0.79 (95% CI: 0.75–0.83). The forest plot summarizing individual study estimates and the pooled effect is shown in Fig. 3.

Substantial between-study heterogeneity was observed, with an estimated τ^2^ of 0.141 and an I^2^ of 73.14%. Cochran's Q test confirmed statistically significant heterogeneity (Q = 127.65; p-value <0.001). The Baujat plot (Supplementary Fig. 1) suggested that the study of *Zhao* et al. contributed disproportionately to the overall heterogeneity and influence on the pooled estimate, as indicated by a relatively high squared Pearson residual and influence value [61]. A sensitivity analysis was performed excluding this study and a reduced heterogeneity (τ^2^ = 0.084; I^2^ = 46.1%; p-value: 0.010) was observed, although moderate heterogeneity persisted. Given that this excess variability was not fully explained by a single study and to avoid potential bias related to post hoc exclusion, it was retained in the primary analysis. The radial (Galbraith) plot (Supplementary Fig. 2) showed that most standardized residuals were distributed within the ±2 boundaries, with no clear extreme outliers. A small number of studies showed moderate deviation, but none exceeded conventional thresholds for major concern. Visual inspection of the funnel plot suggested a degree of asymmetry (p-value: 0.029), potentially consistent with small-study effects or publication bias (Supplementary Fig. 3). Results of the Egger-type regression test should be interpreted cautiously given the limited number of included cohorts and the substantial between-study heterogeneity. PROBAST analysis highlighted that the main limitations were observed in the analysis domain, primarily driven by retrospective study designs, limited sample sizes, and lack of external validation. Regarding applicability, most studies presented some concerns, mainly related to the predictors used. In particular, models based on radiomics, genomics, or complex ML pipelines showed reduced clinical applicability (Supplementary Fig. 4).

### 3.4 Subgroup analyses

Eleven studies (55.0%) evaluated unimodal AI models [23,32,37,46,47,58,60,61,76,77], while 9 studies (45.0%) implemented multimodal frameworks [26,27,29,31,38,42,43,48,52]. The pooled AUC was 0.77 (95% CI: 0.70–0.82) for unimodal models and 0.80 (95% CI: 0.75–0.85) for multimodal models, with no statistically significant difference (p-value: 0.337). Heterogeneity was substantial among unimodal studies (I^2^ = 81.6%) and more moderate among multimodal studies (I^2^ = 36.3%). Eight studies (40.0%) developed models without radiomics features [23,32,43,46,60,61,77], whereas 12 (60.0%) incorporated radiomics-based inputs [26,27,29,31,37,38,42,47,48,52,58,76]. Models including radiomics data showed a numerically higher pooled AUC (0.81, 95% CI: 0.76–0.85), compared with studies not incorporating radiomics features (0.75, 95% CI: 0.68–0.81) although this difference did not reach statistical significance (p-value: 0.109). Moderate -substantial heterogeneity was observed in both subgroups (I^2^ = 50.8% and 77.1%, respectively). Similarly, 14 studies (70.0%) relied exclusively on pre-treatment data [23,26,29,31,32,38,42,43,46,47,58,61,77], while 6 (30.0%) incorporated longitudinal information acquired during treatment [27,37,48,52,60,76]. The pooled AUC was 0.77 (95% CI: 0.72–0.81) for model based exclusively on pre-treatment data and 0.82 (95% CI: 0.75–0.87) for models including longitudinal data, without a statistically significant difference (p = 0.216). Notably, heterogeneity was lower among studies using longitudinal data (I^2^ = 35.4%) compared with baseline-only models (I^2^ = 75.7%). Finally, the impact of incorporating clinical variables was evaluated: 9 studies (45.0%) developed AI models without clinical inputs [32,37,[46], [47], [48],^58^,^60^,^76^,^77^], while 11 (55.0%) included clinical data as part of the feature set [23,26,27,29,31,38,42,52,60,61]. The pooled AUC was 0.80 (95% CI: 0.74–0.85) in the absence of clinical variables and 0.78 (95% CI: 0.72–0.82) when clinical data were included; this difference was not statistically significant (p-value: 0.537). Substantial heterogeneity persisted in both subgroups (I^2^ = 57.7% and 74.3%, respectively), indicating that factors beyond individual data modalities contribute to the variability in predictive performance across studies. Although some subgroups showed numerically lower τ^2^ and I^2^ estimates, the wide confidence intervals and limited number of studies in several categories preclude firm conclusions regarding differential heterogeneity across study characteristics. Detailed data are reported in Supplementary Table 3–4.

### 3.5 Meta-regression and study heterogeneity

Meta-regression analyses showed limited reduction in between-study heterogeneity across investigated moderators. The between-study variance in the null model was τ^2^ = 0.141. The inclusion of radiomics (τ^2^ = 0.1256; pseudo-R^2^ = 10.9%), longitudinal data (τ^2^ = 0.1286; pseudo-R^2^ = 8.7%), unimodal design (τ^2^ = 0.1297; pseudo-R^2^ = 8.0%), and the combination of radiomic and clinical data (τ^2^ = 0.1254; pseudo-R^2^ = 11.1%) was associated with modest reductions in τ^2^, suggesting that these factors may partially account for between-study variability. Subtype-specific internal validation showed a more limited impact on heterogeneity (pseudo-R^2^ = 3.8%). Moreover, the inclusion of clinical data, computational pathology features, genomic data, or DL approaches was associated with no reduction—or even slight increases—in residual heterogeneity (negative pseudo-R^2^ values), indicating that these moderators did not explain variability in discriminative performance. Overall, the proportion of heterogeneity explained by individual moderators was small (<15%), suggesting that between-study variability in AUC is likely influenced by multiple interacting methodological and population-level factors rather than by any single study characteristic.

## 4 Discussion

Predicting which patients with eTNBC are most likely to achieve benefit from NAST remains a relevant clinical challenge. Several studies using AI approaches, including both conventional ML methods and DL architectures, have reported encouraging results for prediction of pCR [18,81,82]. In parallel, integrative approaches combining clinical and biological variables with genomic, metabolomic, radiological and histopathological data have been proposed as a means to improve predictive accuracy by capturing complementary aspects of tumor phenotype and behaviour [81,83,84]. Despite these advances, it remains unclear which modeling strategies and input modalities are most informative specifically in the eTNBC setting [61].

To our knowledge, the present study represents the first systematic review and meta-analysis to provide a comprehensive qualitative and quantitative synthesis of the evidence on AI-based models for predicting pCR following NAST in eTNBC. Our findings complement and extend those of *Krasniqi* et al., who reported promising performance of multimodal DL models for pCR prediction in early BC, but were unable to perform a meta-analysis due to substantial heterogeneity across studies [18]. By pooling data from 20 cohorts, we derived an exploratory estimate of average model performance, with a pooled AUC of 0.79 (95% CI: 0.75–0.83), supporting further investigation focusing on AI-based predictive approaches to capture clinically relevant signals associated with treatment sensitivity in this biologically aggressive subtype. Beyond overall discrimination, a key issue for clinical translation is whether AI adds predictive value beyond established clinicopathologic factors, including tumour size, nodal status, histological grade, Ki-67, and tumor-infiltrating lymphocytes (TILs). Several included studies addressed this point by comparing AI-based with conventional clinicopathologic models. For example, *Li et al.* reported that a DL-derived pCR-score from histopathological images improved the AUC of a baseline model from 0.839 to 0.890 [50], while *Zeng et al.* and *Mao et al.* found that multimodal approaches outperformed non-AI-based models [39,66].

Subgroup analyses provided additional insights into the relative contribution of specific data types and modeling strategies. Radiomics-based models showed numerically higher performance compared with non-radiomics approaches, although differences were not statistically significant. Radiological imaging, especially breast MRI, represents the most extensively investigated data modality for eTNBC treatment response prediction [61]. Several meta-analyses focusing on MRI-derived radiomic features have reported favorable predictive performance for pCR following NAST, with pooled AUC values ranging from 0.78 to 0.85 [[85], [86], [87]] Similarly, US-based radiomics has shown encouraging results: a recent meta-analysis including eight studies reported pooled AUC values of 0.86 [88,89]. Beyond data modality, the timing of data acquisition emerged as a key determinant of predictive performance. The majority of AI tools for disease monitoring rely on a single time point, missing the possibilities to exploit temporal self-supervision for disease progression [88,90]. Furthermore, information acquired at different timepoints can contribute to a better characterization of longitudinal changes of the primary tumors and their response to treatment. In our study, models incorporating longitudinal data during treatment demonstrated numerically improved discrimination and notably lower heterogeneity, suggesting that dynamic, treatment-informed features may be more robust than baseline-only data. This observation is biologically plausible and consistent with prior evidence indicating that early treatment-induced changes in tumor cellularity, edema, and perfusion—captured through serial imaging—may reflect chemosensitivity more accurately than static baseline morphology alone [91]. Multimodal models are conceptually attractive because they aim to integrate large-scale phenotypic information from imaging, high-resolution biological detail from pathology or omics, and contextual clinical variables [[92], [93], [94]]. However, in the present analysis, multimodal approaches did not consistently outperform unimodal models, indicating that simply increasing data complexity does not guarantee better performance, and that data quality, harmonization, and availability remain critical [95].

Beyond subgroup findings, our study is characterized by substantial heterogeneity (I^2^ = 73.14, p-value <0.0001), underscoring that AI-based prediction of pCR should not be regarded as a single, uniform intervention. Rather, it reflects a diverse set of analytical pipelines differing in data input modality, timing of acquisition, model architecture, and validation rigor, with variability in performance partly driven by identifiable technical, clinical, and biological factors. These findings are consistent with the PROBAST assessment, which identified the analysis domain as the main source of bias, largely due to methodological heterogeneity, limited external validation, and variability in model development strategies. From a technical perspective, radiomics-based approaches are particularly sensitive to upstream processing steps, including tumor segmentation accuracy, image acquisition parameters, and scanner- or protocol-related variability. Feature stability and reproducibility across institutions and imaging platforms remain critical challenges, as even subtle differences in acquisition or reconstruction can significantly alter handcrafted feature distributions and, in turn, model performance [92,96,97]. In contrast, DL models—especially end-to-end architectures— reduce reliance on explicit feature engineering but introduce alternative sources of vulnerabilities. These include susceptibility to shortcut learning, whereby models exploit spurious correlations unrelated to underlying tumor biology, and limited robustness to domain shift across institutions or imaging platforms [98]. Such effects may lead to overly optimistic performance during internal validation while impairing transferability to external cohorts. Imaging modality itself represents an additional and important dimension of technical heterogeneity. MRI-based model performance is closely tied to standardized acquisition protocols, contrast timing, and segmentation strategies, while US- and PET/CT-based approaches are subject to higher operator dependence, lower spatial resolution, or variability in reconstruction algorithms [[99], [100], [101]]. Pathology-based and computational pathology models are influenced by variability in staining protocols, tissue processing, slide scanners, magnification, and image resolution. Differences in stain normalization, tissue quality control, annotation, and segmentation methods, together with sampling bias from core biopsies and intratumoral heterogeneity, may further affect feature extraction and model reproducibility. These issues are particularly relevant in eTNBC, where features such as TILs density, stromal architecture, and tumour–immune spatial relationships may be associated with treatment response. However, adherence to standardized pathology guidelines, including TILs assessment recommendations, was inconsistently reported [102,103]. An additional source of heterogeneity arises from the incomplete and often selective availability of multimodal data. In real-world settings, not all patients undergo standardized MRI, digital pathology, and molecular profiling with comparable quality and timing, meaning that multimodal cohorts may represent a clinically enriched subset rather than the target population. This selection bias, together with missing or variable data quality, can limit generalizability and may partly explain why multimodal models do not consistently outperform simpler approaches [104,105]. Overall, these technical factors highlight that differences in model performance are not solely attributable to algorithmic choice, but rather emerge from complex interactions between data acquisition, preprocessing pipelines, and model architecture. The lack of standardized workflows across studies remains a key barrier to reproducibility and external validation, ultimately limiting the clinical translation of AI-based predictive models [106,107]. In addition to the technical sources of variability discussed above, clinical definitions and treatment-related factors contribute substantially to the heterogeneity observed across studies. While the pCR endpoint was consistently defined as ypT0/is ypN0 across included studies, other clinical and biological definitions remained heterogeneous. Even within TNBC, the definition of “triple-negative” is not fully uniform: some cohorts adopt estrogen and progesterone receptor thresholds of <1%, whereas others use <10%, and HER2 testing algorithms have evolved over time [108]. These differences are clinically meaningful, as relatively small shifts in receptor cutoffs may alter the underlying biological composition of the study population, including immune infiltration, proliferation indices, and other tumor characteristics that AI models may indirectly capture. As a result, variability in TNBC definition can influence both model training and apparent predictive performance, further complicating cross-study comparisons and external validation [109,110]. Furthermore, a major clinical and temporal source of heterogeneity is represented by the rapid evolution of NAST strategies for eTNBC. Over the past decade, neoadjuvant schemes have evolved substantially, with the increasing incorporation of platinum-based chemotherapy and the introduction of immune checkpoint inhibitors for high-risk eTNBC [111]. KEYNOTE-522 established pembrolizumab combined with chemotherapy as a new therapeutic standard by demonstrating improvements in pCR, event-free survival, and, more recently, overall survival [7,112]. However, most AI studies included in our review were conducted before the widespread adoption of immunotherapy, and several did not provide detailed information on the administered NAST regimens. The interpretation and generalizability of these findings is further limited by the marked heterogeneity of chemotherapy regimens adopted in the pre-platinum and pre-immunotherapy eras. In our dataset, only two studies included pembrolizumab [38,64], while five reported carboplatin as part of the chemotherapy backbone [35,47,56,64,76]. This treatment-era heterogeneity is clinically relevant because models trained on historical chemotherapy-only cohorts may not be directly transportable to contemporary chemoimmunotherapy-treated populations. While carboplatin mainly modifies the cytotoxic backbone, immunotherapy may alter both baseline predictors of response and on-treatment dynamics, including immune infiltration, stromal remodeling, edema, and imaging or pathological features related to treatment-induced immune activation. As a result, the relationship between input features and pCR may differ across treatment eras, potentially leading to performance degradation when models are applied outside the therapeutic context in which they were developed [113]. However, some of the observed variability may reflect differences in real-world clinical practice, where neoadjuvant treatment approaches for eTNBC are not yet fully standardized across institutions and healthcare systems [114,115].

Despite these limitations, this review and meta-analysis provide a comprehensive synthesis of available evidence, highlighting both the potential of AI and the current key gaps. Through the identification of data modalities and modeling strategies associated with more robust performance, (radiomic-based models and/or longitudinal data integration), it may help guide future research and promote greater methodological harmonization. Validation strategy and reporting remain key limitations. External validation was infrequent, and many studies relied on cross-validation without strict temporal or site separation, which can inflate performance estimates, especially when preprocessing is not confined to training folds in high-dimensional pipelines. Evidence of publication bias further suggests overrepresentation of small studies with optimistic results. These issues underscore the need for standardized reporting, pre-registration when feasible, and multi-institutional benchmarking with locked pipelines alongside consistent external validation. Future studies should prioritize prospective, multicenter validation and extend AI applications beyond pCR toward clinically meaningful endpoints, while adhering to emerging reporting and methodological standards such as TRIPOD-AI [116] and CONSORT-AI [117] to improve transparency, reproducibility, and comparability across studies. Ultimately, well-validated AI tools may support more personalized treatment strategies in eTNBC, including treatment escalation, de-escalation, and optimization of immunotherapy use.

## 5 Conclusions

This systematic review and meta-analysis suggests that AI-based models can achieve good average discrimination for predicting pCR after NAST in eTNBC. At the same time, substantial heterogeneity and inconsistent methodology currently limit generalizability and clinical use. Beyond providing an overall pooled estimate, this study helps identify study features that may be associated with more promising performance within a fragmented literature. Progress in this area will depend on standardized reporting, stronger external validation, and prospective multicenter evaluation so that more robust and clinically applicable predictive models can be developed.

## Ethical approval and consent to participate

Not applicable.

## Consent for publication

Not applicable.

## Authors' contributions

AC, FM, and AM contributed to the conceptualization of the study. Data acquisition and collection were performed by AC, FM, MS, and FF, while study selection was carried out by AC, FF, FM and AA. Methodology was developed by AC, FM, MS, and FF. Formal analysis was conducted by FF, AC, and FM, and data visualization was performed by FF and AC. The initial draft of the manuscript was prepared by AC, FM, and MS. AM supervised the study. Critical revision and editing of the manuscript were undertaken by all the authors.

## Funding

Not applicable.

## CRediT authorship contribution statement

**Andrea Carlini:** Conceptualization, Data curation, Formal analysis, Investigation, Methodology, Visualization, Writing – original draft, Writing – review & editing. **Flavia Foca:** Data curation, Formal analysis, Methodology, Software, Visualization, Writing – review & editing. **Marianna Sirico:** Data curation, Methodology, Writing – original draft, Writing – review & editing. **Alberto Farolfi:** Writing – review & editing. **Chiara Casadei:** Writing – review & editing. **Caterina Gianni:** Writing – review & editing. **Michela Palleschi:** Writing – review & editing. **Nicola Gentili:** Writing – review & editing. **Marita Mariotti:** Writing – review & editing. **Giandomenico Di Menna:** Writing – review & editing. **Alice Andalò:** Data curation, Writing – review & editing. **Paolo De Angelis:** Writing – review & editing. **Olga Serra:** Writing – review & editing. **Simone Sabbioni:** Writing – review & editing. **Giulia Miserocchi:** Writing – review & editing. **Daniela Montanari:** Writing – review & editing. **Lorenzo Cecconetto:** Writing – review & editing. **Samanta Sarti:** Writing – review & editing. **Antonino Musolino:** Resources, Supervision, Validation, Writing – review & editing. **Filippo Merloni:** Conceptualization, Data curation, Formal analysis, Investigation, Methodology, Project administration, Writing – original draft, Writing – review & editing.
