# Supplementary material for: Artificial intelligence for predicting pathological complete response to neoadjuvant therapy in triple-negative breast cancer: A systematic review and meta-analysis
Source: Breast. 2026 Jul 2;89:104861. doi: 10.1016/j.breast.2026.104861 (PMC13355824; doi:10.1016/j.breast.2026.104861)
Supplement: Multimedia component 1 [file mmc1.docx]

**Supplementary Table 1. Detailed search strategy and query strings**

| ***PubMed*** |
| --- |
| ("Breast Neoplasms"[Mesh]  OR "breast cancer"[tiab]  OR "breast carcinoma"[tiab]  OR "breast neoplasm*"[tiab])  AND  ("Neoadjuvant Therapy"[Mesh]  OR "neoadjuvant chemotherapy"[tiab]  OR "neoadjuvant treatment"[tiab]  OR "neoadjuvant immunotherapy"[tiab]  OR "preoperative therapy"[tiab]  OR "primary systemic therapy"[tiab]  OR NAC[tiab])  AND  ("Artificial Intelligence"[Mesh]  OR "Machine Learning"[Mesh]  OR "Deep Learning"[Mesh]  OR "machine learning"[tiab]  OR "deep learning"[tiab]  OR radiomics[tiab]  OR "neural network*"[tiab]  OR "computational pathology"[tiab]  OR "computer-aided diagnosis"[tiab]  OR "predictive model*"[tiab]  OR transformer*[tiab]  OR GPT[tiab]  OR BERT[tiab]  OR "large language model*"[tiab]  OR autoencoder*[tiab]  OR "generative adversarial network*"[tiab]  OR GAN[tiab]  OR "federated learning"[tiab]  OR "transfer learning"[tiab]  OR "ensemble learning"[tiab]  OR "self-supervised learning"[tiab]  OR "multimodal AI"[tiab])  AND  ("treatment response"[tiab]  OR "pathological complete response"[tiab]  OR pCR[tiab]  OR "residual cancer burden"[tiab]  OR RCB[tiab]  OR "treatment efficacy"[tiab]) |
| ***Embase*** |
| ('breast tumor'/exp OR 'breast cancer':ti,ab OR 'breast carcinoma':ti,ab OR 'breast neoplasm':ti,ab)  AND  ('neoadjuvant therapy'/exp OR 'neoadjuvant chemotherapy':ti,ab OR 'neoadjuvant treatment':ti,ab OR NAC:ti,ab OR 'primary systemic therapy':ti,ab OR 'neoadjuvant immunotherapy':ti,ab OR 'preoperative therapy':ti,ab)  AND  ('artificial intelligence'/exp  OR 'machine learning'/exp  OR 'deep learning'/exp  OR 'radiomics'/exp  OR 'neural network'/exp  OR 'computer aided diagnosis'/exp  OR 'computational pathology':ti,ab  OR 'predictive modeling':ti,ab  OR 'machine learning':ti,ab  OR 'deep learning':ti,ab  OR radiomics:ti,ab  OR 'neural network*':ti,ab  OR 'computer aided diagnosis':ti,ab  OR 'computational pathology':ti,ab  OR 'predictive model*':ti,ab  OR transformer*:ti,ab  OR GPT:ti,ab  OR BERT:ti,ab  OR 'large language model*':ti,ab  OR autoencoder*:ti,ab  OR 'generative adversarial network*':ti,ab  OR GAN:ti,ab  OR 'federated learning':ti,ab  OR 'transfer learning':ti,ab  OR 'ensemble learning':ti,ab  OR 'self supervised learning':ti,ab  OR 'multimodal AI':ti,ab  )  AND  ('treatment response':ti,ab  OR 'pathological complete response':ti,ab  OR pCR:ti,ab  OR 'residual cancer burden':ti,ab  OR RCB:ti,ab  OR 'treatment efficacy':ti,ab) |
| ***Cochrane CENTRAL*** |
| ("breast cancer" OR "breast carcinoma" OR "breast neoplasm*"):ti,ab,kw  AND  ("neoadjuvant therapy" OR "neoadjuvant chemotherapy" OR "neoadjuvant treatment"  OR "neoadjuvant immunotherapy" OR "preoperative therapy" OR "primary systemic therapy" OR NAC):ti,ab,kw  AND  ("artificial intelligence" OR "machine learning" OR "deep learning"  OR radiomics OR "neural network*" OR "computational pathology"  OR "predictive model*"):ti,ab,kw  AND  ("treatment response" OR "pathological complete response" OR pCR  OR "residual cancer burden" OR RCB):ti,ab,kw |

**Supplementary Table 2. Leave-one-out sensitivity analysis of pooled AUC estimates**

| Removed cohort | Pooled AUC (95%CI) | I² | τ² |
| --- | --- | --- | --- |
| Aswolinskiy et al. (1) | 0.79 (0.75-0.83) | 74.815 | 0.152 |
| Aswolinskiy et al. (2) | 0.79 (0.75-0.83) | 73.456 | 0.139 |
| Banerjee et al. | 0.78 (0.74-0.82) | 73.882 | 0.141 |
| Cai et al | 0.79 (0.75-0.83) | 74.098 | 0.147 |
| Du Terrail et al. | 0.79 (0.75-0.82) | 74.857 | 0.152 |
| Fisher et al. | 0.78 (0.74-0.82) | 70.083 | 0.145 |
| Guo et al. | 0.78 (0.74-0.82) | 73.663 | 0.148 |
| Hatamikia et al | 0.78 (0.74-0.82) | 73.670 | 0.139 |
| Huang et al. | 0.78 (0.74-0.82) | 70.105 | 0.128 |
| Lin et al | 0.78 (0.74-0.82) | 73.521 | 0.137 |
| Orozco et al. | 0.79 (0.75-0.83) | 74.393 | 0.153 |
| Park et al. | 0.78 (0.74-0.82) | 73.299 | 0.136 |
| Qi et al. | 0.79 (0.75-0.82) | 74.807 | 0.148 |
| Ramtohul et al. | 0.78 (0.74-0.82) | 72.874 | 0.137 |
| Sturm et al. | 0.79 (0.75-0.83) | 74.231 | 0.145 |
| Xie et al. | 0.79 (0.75-0.83) | 71.178 | 0.132 |
| Zhang et al. | 0.78 (0.74-0.82) | 73.006 | 0.136 |
| Zhao et al. | 0.80 (0.76-0.83) | 46.400 | 0.084 |
| Lin et al. (2) | 0.79 (0.75-0.83) | 74.682 | 0.154 |
| Mohamed et al. | 0.79 (0.74-0.82) | 74.767 | 0.149 |

**Supplementary Table 3. Detailed characterization of pathology-related and computational pathology studies among 58 studies considered for qualitative analysis.**

| **Study** | **Data input** | **Methodology** | **Staining** | **Segmentation** | **TILs / TME assessment** |
| --- | --- | --- | --- | --- | --- |
| *Aswolinskiy, 2023* | Pretreatment biopsy WSIs | WSI-based DL | H&E | Automated segmentation of tumour, stroma, lymphocytes and other tissue compartments; mitosis detection | Yes:  cTILs, lymphocyte-to-tumour ratio, inflamed tumour ratio, mitotic rate |
| *Bhattarai,*  *2024* | Pretreatment biopsy WSIs  (three serial tissue slides) | WSI-based ML with cell-level analysis | H&E  IHC Ki67 IHC pH3 | Manual annotations with Mask R-CNN-based detection of tumour cells, TILs and IHC-positive cells | Yes:  tTILs, sTILs, tumour cells, Ki67, pH3 |
| *Du Terrail, 2023* | Pretreatment biopsy WSIs + clinical-biological data | WSI-based DL  (federated learning) | H&E | Weakly supervised federated learning; no conventional ROI annotation | Yes:  TILs and histological variables considered; no explicit TME segmentation pipeline |
| *Duanmu, 2022* | Pretreatment biopsy WSIs  (three serial tissue slides) | WSI-based DL with spatial attention | H&E  IHC Ki67 IHC PHH3 | Mask R-CNN-based tumour cell identification with IHC biomarker quantification and spatial attention | Indirect:  proliferation- and mitosis-related tumour regions |
| *Fisher, 2024* | Pretreatment biopsy WSIs | WSI-based ML with spatial graph analysis | H&E | Manual annotation of histological classes with tile-level classification and map generation | Yes:  tTILs, sTILs, tertiary TILs, MVD, PGCCs and necrosis |
| *Hacking, 2023* | Pretreatment biopsy WSIs (tumour and stromal features) + clinical-biological data | WSI-based ML feature quantification | H&E | Automated quantification of tumour and stromal components | Yes:  myxoid, collagenous and immune stromal features |
| *Krishnamurthy, 2023* | Pretreatment biopsy WSIs + clinical-biological data | WSI-based DL with morphometric clustering | H&E | Expert-guided annotation of morphological categories with unsupervised feature extraction | Yes:  tumour-, stroma- and TIL-related morphometric patterns |
| *Li, 2021* | Pretreatment biopsy WSIs | WSI-based DL | H&E | Automated tumour epithelium identification and pCR score generation | No:  sTILs included as comparator, not as primary computational target |
| *Li, 2022 (npj)* | Pretreatment biopsy WSIs + clinical-biological data | WSI-based DL for stromal biomarker analysis | H&E | Epithelium–stroma classification following pathologist-guided ROI segmentation, with stromal tile selection and limited manual intervention | Yes:  stromal morphology, collagen, fibroblasts and sTILs (used as comparator) |
| *Li, 2024* | Pretreatment biopsy WSIs | WSI-based DL | H&E | Pathologist annotation of histological classes with graph construction from label maps | Yes:  immune cells, collagen and spatial TME architecture |
| *Li, 2022 (The Breast)* | Pretreatment biopsy WSIs + clinical-biological data | WSI-based DL with patch-level aggregation | H&E | Automated patch classification and feature aggregation without manual ROI annotation | No:  sTILs used as comparator |
| Mao, 2025 | Pretreatment biopsy WSIs + MRI + clinical-biological data | Multimodal DL integrating WSI, MRI and clinical data | H&E | Automated patch-level classification and feature extraction, followed by multimodal feature integration and attention heatmap exploration | Yes:  immune-related pathways and antitumour microenvironment features explored |
| *Sturm, 2025* | Pretreatment biopsy WSIs | WSI-based DL | H&E | Manual tumour-area annotation with weakly supervised image-level prediction | No |
| *Wang, 2024* | Multiscale WSIs | WSI-based DL (multiscale multitask) | H&E | Foreground segmentation, multiscale patch extraction and attention heatmap generation | Yes:  TILs assessed and immune-related biological associations explored |
| Zeng et al. | ROI-based biopsy WSIs | ROI-based DL with ML classifier | H&E | Representative ROI selection across magnifications with DL-based feature extraction | No:  General tumour and stromal information; no detailed TME quantification |

*Abbreviations*: 16S rDNA, 16S ribosomal DNA; AI, artificial intelligence; cTILs, computational tumor-infiltrating lymphocytes; DL, deep learning; FISH, fluorescence in situ hybridization; H&E, hematoxylin and eosin; HE, hematoxylin and eosin; IHC, immunohistochemistry; Ki67, Kiel-67 proliferation marker; LPS, lipopolysaccharide; Mask R-CNN, Mask Region-based convolutional neural network; mIF, multiplex immunofluorescence; ML, machine learning; MRI, magnetic resonance imaging; MVD, microvessel density; pCR, pathological complete response; pH3/PHH3, phosphohistone H3; PGCCs, polyploid giant cancer cells; ROI, region of interest; RT-qPCR, real-time quantitative polymerase chain reaction; scRNA-seq, single-cell RNA sequencing; SPP1+, secreted phosphoprotein 1-positive; sTILs, stromal tumor-infiltrating lymphocytes; TILs, tumor-infiltrating lymphocytes; TME, tumor microenvironment; tTILs, intratumoral tumor-infiltrating lymphocytes; WSI, whole-slide image.

**Supplementary Table 4. Subgroup meta-analysis of AUC and test for between-group differences**

| Subgroup | N° of studies by subgroup | AUC (95%CI) | p-value for moderator test |
| --- | --- | --- | --- |
|  |  |  |  |
| Mixed validation cohort | 8 (40.0) | 0.76 (0.69;0.82) | 0.340 |
| Only TNBC validation cohort | 12 (60.0) | 0.80 (0.75;0.84) |  |
|  |  |  |  |
| Multimodal | 11 (55.0) | 0.80 (0.75;0.85) | 0.337 |
| Unimodal | 9 (45.0) | 0.77 (0.70;0.82) |  |
|  |  |  |  |
| No radiomics data | 8 (40.0) | 0.75 (0.68;0.81) | 0.109 |
| Presence of radiomics data | 12 (60.0) | 0.81 (0.76;0.85) |  |
|  |  |  |  |
| Only baseline data | 14 (70.0) | 0.77 (0.72;0.81) | 0.216 |
| Presence of longitudinal data | 6 (30.0) | 0.82 (0.75;0.87) |  |
|  |  |  |  |
| Absence of clinical data | 9 (45.0) | 0.80 (0.74;0.85) | 0.537 |
| Utilization of clinical data | 11 (55.0) | 0.78 (0.72;0.82) |  |
|  |  |  |  |
| Absence of computational pathology | 15 (75.0) | 0.80 (0.75;0.84) | 0.468 |
| Utilization of  computational pathology | 5 (25.0) | 0.76 (0.67;0.84) |  |
|  |  |  |  |
| Absence of genomic data | 17 (85.0) | 0.78 (0.74;0.82) | 0.373 |
| Presence of  genomic data | 3 (15.0) | 0.83 (0.72;0.90) |  |
|  |  |  |  |
| Combination of radiomic and clinical data | 7 (35.0) | 0.82 (0.75;0.87) | 0.212 |
| Other studies | 13 (65.0) | 0.77 (0.72;0.81) |  |
|  |  |  |  |
| ML | 18 (90.0) | 0.79 (0.75;0.83) | 0.409 |
| DL +/- ML | 2 (10.0) | 0.73 (0.54;0.86) |  |
|  |  |  |  |
| Study with < 60 pts in validation set | 9 (45.0) | 0.79 (0.71-0.85) | 0.969 |
| Study with >= 60 pts in validation set | 11 (55.0) | 0.79 (0.74-0.83) |  |

**Supplementary Table 5. Between-study heterogeneity across subgroups**

| Subgroup | N° of studies by subgroup | τ² (95%CI) | I² (95%CI) |
| --- | --- | --- | --- |
|  |  |  |  |
| Mixed validation cohort | 8 (40.0) | 0.227 (0.049;1.198) | 77.4 (42.9;96.8) |
| Only TNBC validation cohort | 12 (60.0) | 0.074 (0.00;0.438) | 43.6 (0.0;83.5) |
|  |  |  |  |
| Multimodal | 11 (55.0) | 0.062 (0.000;0.679) | 36.3 (0.0;86.2) |
| Unimodal | 9 (45.0) | 0.189 (0.044;1.132) | 81.6 (50.9;96.4) |
|  |  |  |  |
| No radiomics data | 8 (40.0) | 0.135 (0.031;1.027) | 77.1 (43.5;96.2) |
| Presence of radiomics data | 12 (60.0) | 0.116 (0.004;0.593) | 50.8 (3.8;84.1) |
|  |  |  |  |
| Only baseline data | 14 (70.0) | 0.159 (0.045-0.789) | 75.7 (46.9-93.9) |
| Presence of longitudinal data | 6 (30.0) | 0.053 (0.000-0.625) | 35.4 (0.0-86.6) |
|  |  |  |  |
| Absence of clinical data | 9 (45.0) | 0.136 (0.006;0.926) | 57.7 (5.8;90.3) |
| Utilization of clinical data | 11 (55.0) | 0.156 (0.041;0.798) | 74.3 (43.3;93.7) |
|  |  |  |  |
| Absence of computational pathology | 15 (75.0) | 0.182 (0.052;0.627) | 75.7 (46.9;91.5) |
| Utilization of  computational pathology | 5 (25.0) | 0.087 (0.000;1.167) | 49.7 (0.0;92.9) |
|  |  |  |  |
| Absence of genomic data | 17 (85.0) | 0.143 (0.043;0.480) | 74.6 (46.8;90.8) |
| Presence of  genomic data | 3 (15.0) | 0.218 (0.000;14.356) | 56.4 (0.0;98.8) |
|  |  |  |  |
| Combination of radiomic and clinical data | 7 (35.0) | 0.059 (0.000;1.343) | 36.8 (0.0;93.0) |
| Other studies | 13 (65.0) | 0.151 (0.038;0.664) | 75.2 (43.5;93.0) |
|  |  |  |  |
| ML | 18 (90.0) | 0.153 (0.049;0.518) | 75.9 (50.6;91.4) |
| DL +/- ML | 2 (10.0) | 0.000 (0.000;60.896) | 0.0 (0.0;99.7) |
|  |  |  |  |
| Study with < 60 pts in validation set | 9 (45.0) | 0.108 (0.000;1.599) | 32.3 (0.0;87.6) |
| Study with >= 60 pts in validation set | 11 (55.0) | 0.157 (0.049;0.520) | 82.4 (59.4;93.9) |
|  |  |  |  |

**Supplementary Figure 1. Baujat plot**


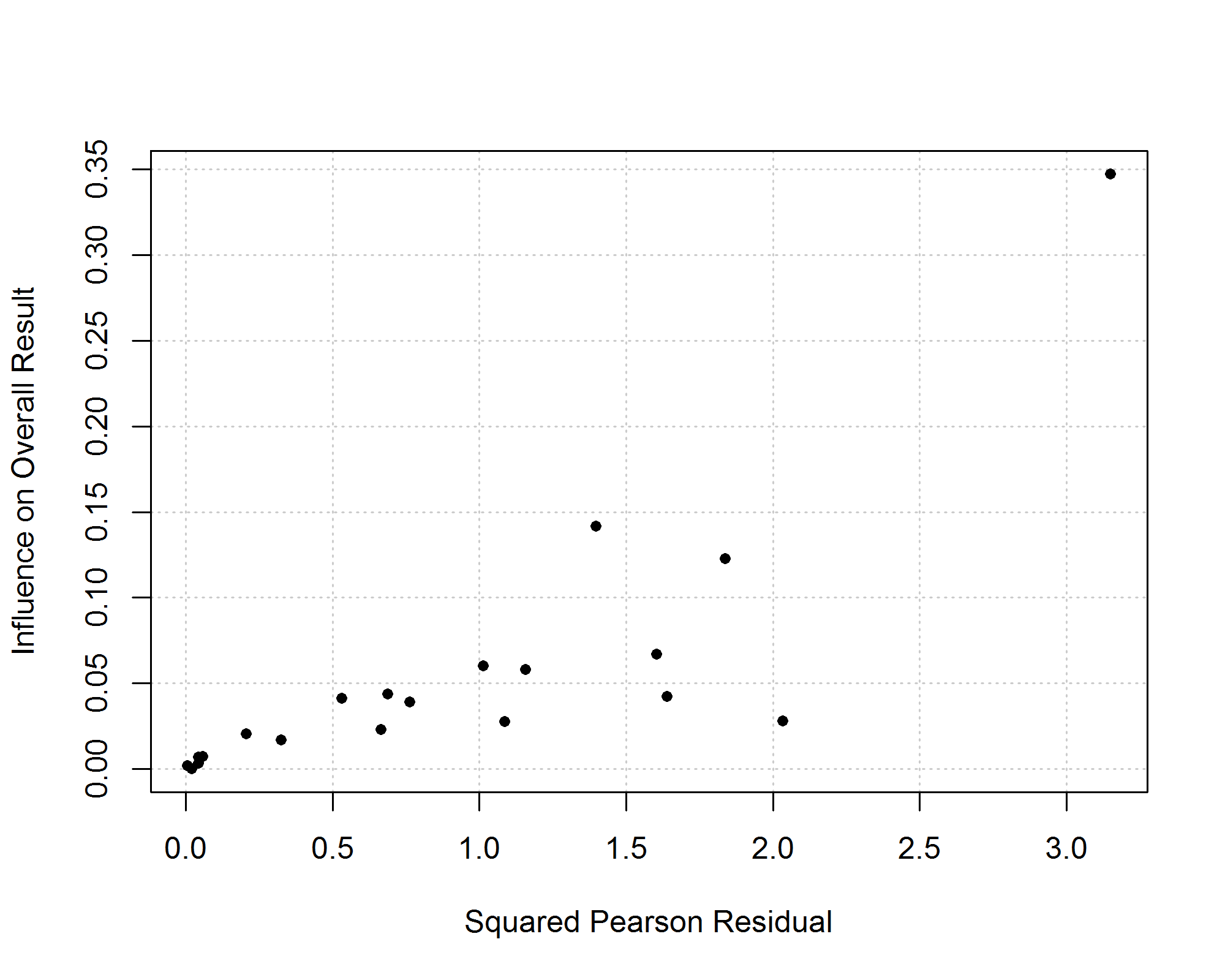


**Supplementary Figure 2. Radial plot**


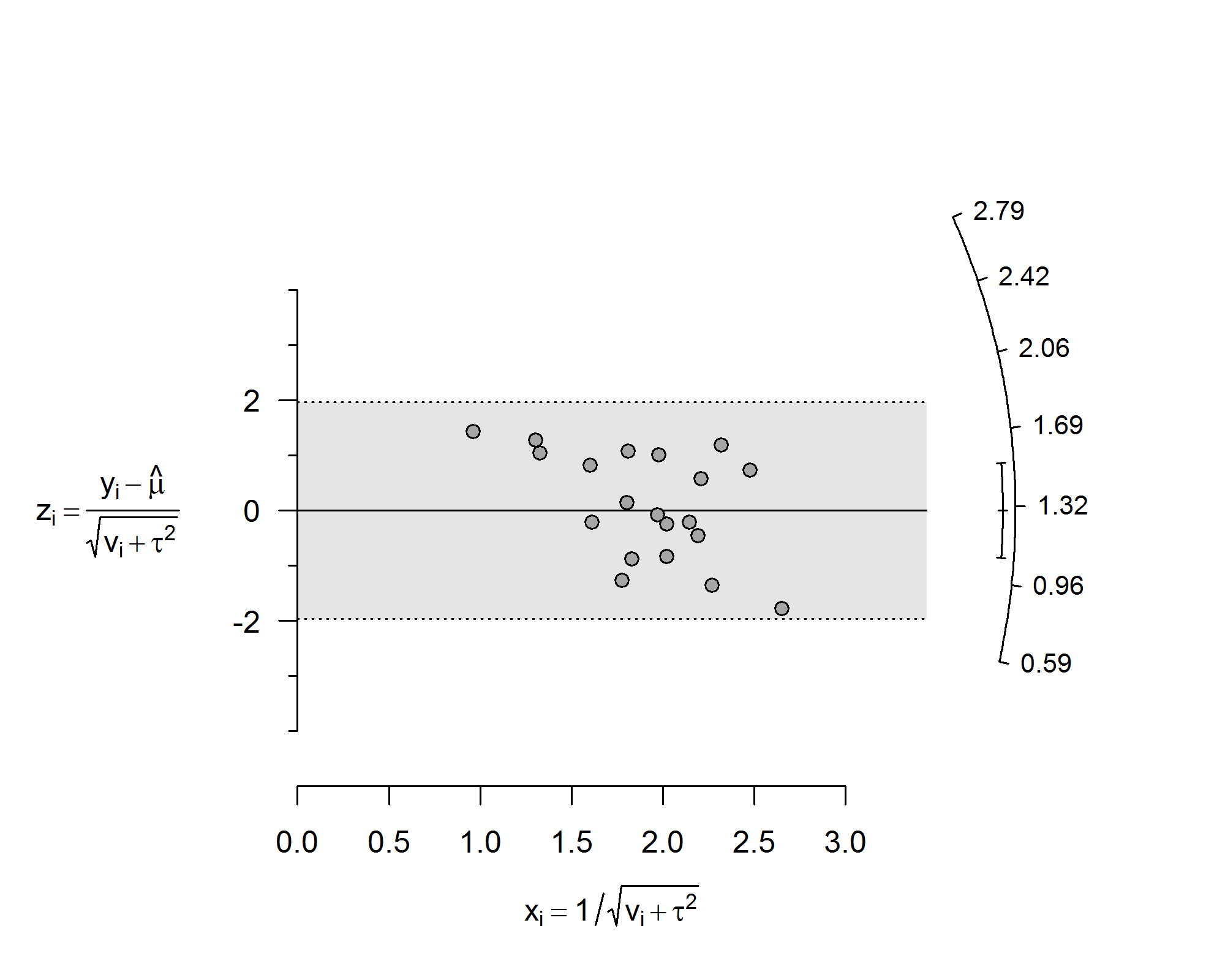


Supplementary results: ***Assessment of publication bias***

Potential publication bias was evaluated visually using funnel plots and formally assessed through statistical testing for funnel plot asymmetry. Visual inspection of the funnel plot suggested asymmetry, with a relative overrepresentation of small studies reporting higher AUC values. This finding was supported by a statistically significant test for asymmetry (p = 0.026), indicating the presence of small-study effects and/or publication bias. Given the limited number of included cohorts, formal tests for funnel plot asymmetry should be interpreted cautiously due to reduced statistical power. These results suggest that studies with less favorable predictive performance may be underrepresented in the published literature. The funnel plot is shown in **Supplementary** **Figure 3**.

**
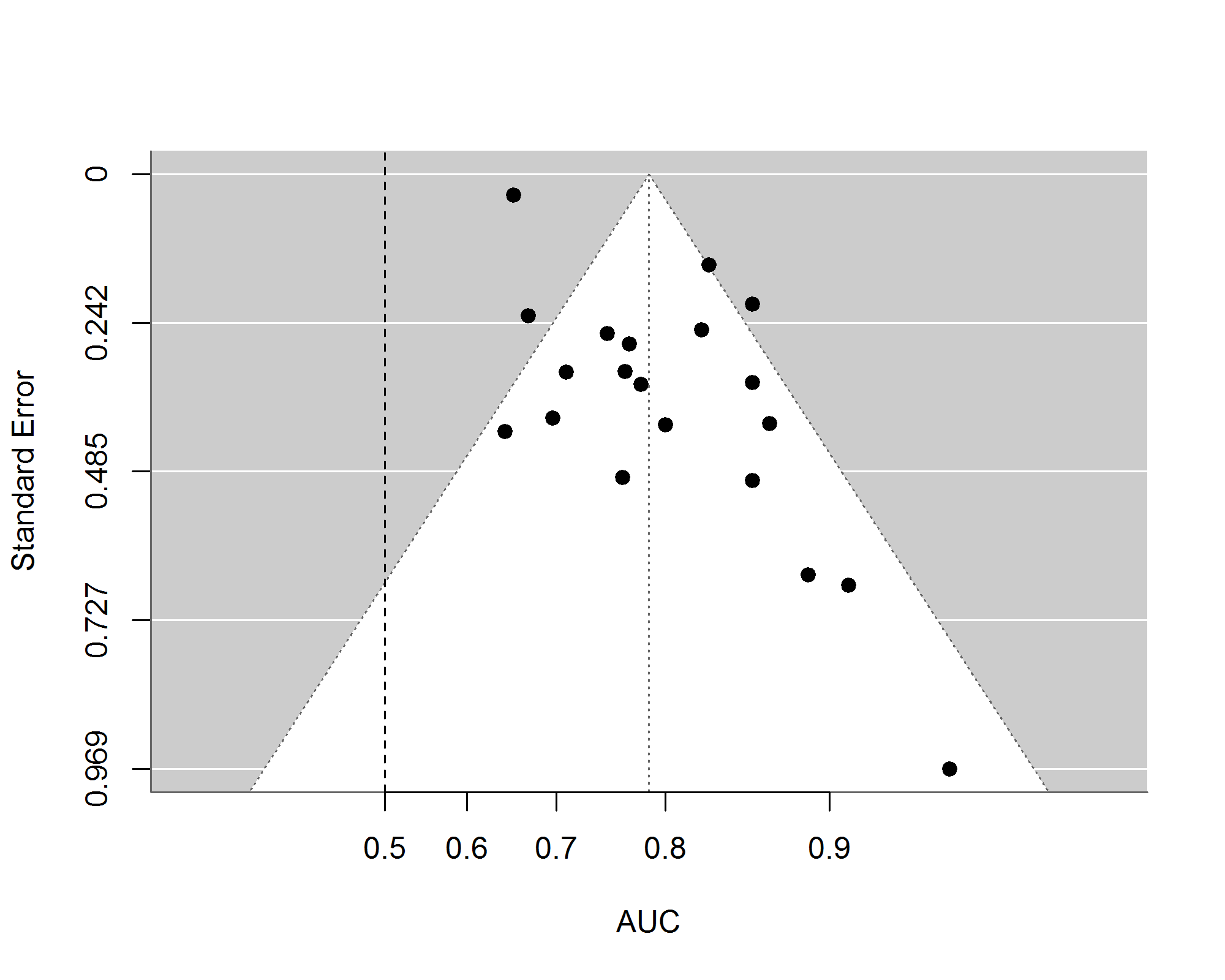
**

**Supplementary figure 3.** Funnel plot for studies included in metanalysis


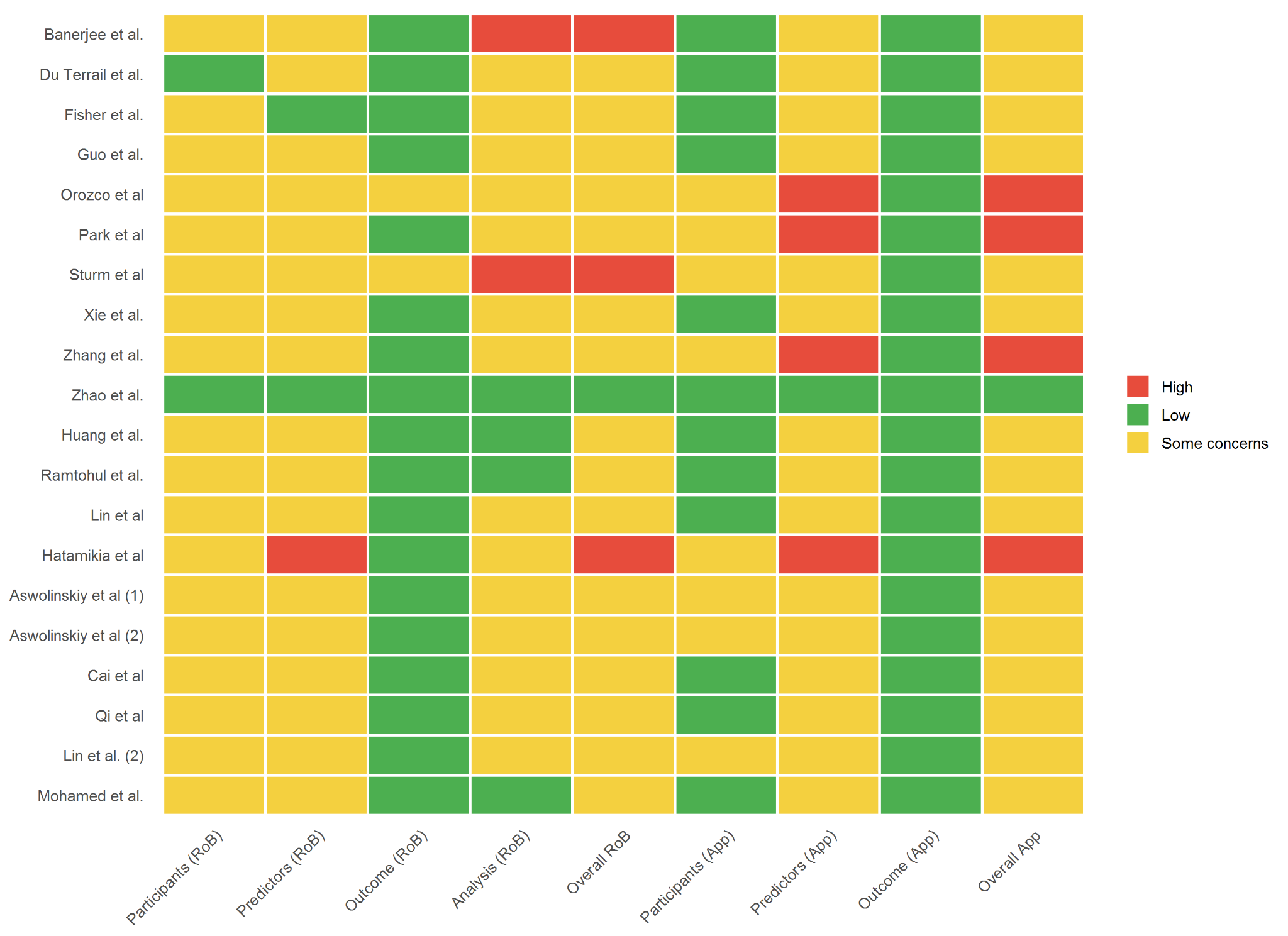


**Supplementary figure 4: Application of PROBAST guidelines results’**
